# Ganglion Cyst of the Temporomandibular Joint: Case Report and a Review of the Literature

**DOI:** 10.3390/dj14100619

**Published:** 2026-09-24

**Authors:** Michał Gontarz, Krzysztof Gąsiorowski, Krystyna Gałązka, Jakub Bargiel, Paweł Szczurowski, Katarzyna Rusek, Kamil Nelke, Grażyna Wyszyńska-Pawelec

**Affiliations:** 1 Department of Cranio-Maxillofacial Surgery, Jagiellonian University Medical College, University Hospital, 2 Jakubowskiego Street, 30-688 Kraków, Poland; krzysztof.gasiorowski@uj.edu.pl (K.G.); jakub.bargiel@uj.edu.pl (J.B.); pawel.szczurowski@uj.edu.pl (P.S.); krusek@su.krakow.pl (K.R.); grazyna.wyszynska-pawelec@uj.edu.pl (G.W.-P.); 2 Department of Pathomorphology, Jagiellonian University Medical College, 31-531 Kraków, Poland; krystyna.galazka@uj.edu.pl; 3 Maxillo-Facial Surgery Ward, EMC Hospital, Pilczycka 144 Street, 54-144 Wrocław, Poland; kamil.nelke@gmail.com

**Keywords:** temporomandibular joint, ganglion cyst, synovial cyst, pseudocyst, temporomandibular disorders

## Abstract

**Background/Objectives**: Ganglion cysts of the temporomandibular joint (TMJ) are rare, benign periarticular pseudocysts that are clinically indistinguishable from the true synovial cyst and frequently mimic parotid or other preauricular tumors, making preoperative diagnosis difficult. The aim of this report is to describe the diagnosis and surgical management of a ganglion cyst of the TMJ and to review the literature on the etiology, clinical presentation, treatment modalities, and outcomes of these lesions. We report a case managed with excision and review the published literature. **Methods**: We describe a 48-year-old woman with a slowly enlarging, painless left preauricular tumor managed by preauricular excision with intraoperative facial-nerve monitoring and provide a narrative review of ganglion and synovial cysts of the TMJ (PubMed/MEDLINE, Scopus and Google Scholar, searched from database inception to July 2026; histologically confirmed cases were extracted and tabulated). **Results**: Computed tomography showed mild condylar hypertrophy and a well-defined, non-enhancing fluid-density lesion (~15 HU; 15 × 10 × 7 mm) lateral to the condyle and abutting the anterior wall of the external auditory canal. The lesion was excised through a preauricular approach, with intraoperative facial-nerve stimulation. Histopathology showed a fibrous-walled cavity devoid of epithelial or synovial cell lining, with negative cytokeratin and CD31 confined to vascular endothelium, confirming a ganglion and excluding a vascular malformation. Facial-nerve function, mouth opening, and occlusion were preserved. Control magnetic resonance imaging at 17 months and clinical follow-up at 21 months showed no recurrence. **Conclusions**: Preauricular excision with facial-nerve preservation is the treatment of choice. Although recurrence is uncommon, documented recurrences several years after excision argue for longer, imaging-based follow-up.

## 1. Introduction

Ganglion and synovial cysts are expansile, fluid-filled lesions that occur most often around the wrist, knee, and foot, with the wrist alone accounting for the majority of cases [1]. Their appearance in the region of the temporomandibular joint (TMJ) is rare, with approximately 50 histologically confirmed cases in the English-language literature [2,3,4]. Two distinct entities are grouped under this heading. The synovial cyst is a true cyst: a herniation or evagination of the synovial membrane that retains a synovial (or, less specifically, an epithelial) lining. The ganglion cyst is a pseudocyst: a fibrous-walled cavity arising from mucoid/myxoid degeneration of periarticular connective tissue that, by definition, lacks any epithelial or synovial cell lining [2,4,5]. Although their pathogenesis and histology differ, the two lesions are clinically indistinguishable, and the terms have historically been used interchangeably, which cause a persistent source of confusion in the literature [2,6].

The etiology of these lesions is not fully understood. Ganglion cysts are generally attributed to mucoid or myxoid degeneration of periarticular collagenous connective tissue, possibly promoted by chronic mechanical irritation, microtrauma, or degenerative joint disease, whereas synovial cysts have additionally been related to increased intra-articular pressure following trauma [3,5]. Both entities occur most often in middle-aged women and usually enlarge slowly, over months to years, as a painless mass.

Because of their location anterior to the tragus, TMJ ganglion and synovial cysts most commonly present as preauricular swelling and are frequently mistaken for a tumor of the parotid gland or for other periauricular masses [2,7,8]. Depending on their exact position and size, they may also cause pain, limited mouth opening, malocclusion, or otological symptoms through dynamic narrowing of the external auditory canal (EAC). Rare cases with intracranial extension and facial-nerve palsy or seizures have been described [9,10,11]. Definitive diagnosis relies on histopathology, and complete surgical excision, usually through a preauricular approach, is the accepted treatment [2,3,12]. Reported treatment modalities range from clinical observation and needle aspiration in asymptomatic patients or those unfit for surgery to complete surgical excision, with aspiration alone carrying a higher rate of recurrence than excision [2,3,12].

The following case report presents a case of a ganglion cyst of the left TMJ in a middle-aged woman, managed by preauricular excision with a favorable functional outcome. The presented case is discussed in the context of previously reported cases identified through a review of the literature.

## 2. Case Presentation

A 48-year-old woman was admitted in November 2024 for evaluation of a mass over the left condylar (preauricular) region. The tumor had first been noticed in May 2024 and had been slowly enlarging. It was painless and had not previously been investigated with imaging. The only relevant comorbidity was bronchial asthma, with spirometry performed in the same year. She reported no pain (0 on a 0–10 numerical rating scale) and no other complaints.

Upon examination, there was a palpable mass in the region of the left mandibular condyle. Mouth opening was within normal limits, and facial nerve function was intact. On the basis of the clinical findings, a tumor of the parotid gland was initially suspected. The patient was therefore referred and investigated for a suspected parotid neoplasm, and computed tomography was the first cross-sectional imaging method used.

Contrast-enhanced computed tomography (CT) demonstrated, at the level of the left TMJ and lateral to the condylar process, a fairly well-circumscribed fluid-attenuation zone (approximately 15 HU) with a single internal septum and no post-contrast enhancement, measuring 15 × 10 × 7 mm, lying in the subcutaneous tissue and abutting the anterior wall of the external auditory canal (Figure 1). The imaging appearance was regarded as indeterminate. However, an origin from the joint could not be excluded, and excision with histological verification was recommended.

Under general anesthesia, the lesion was approached through a standard preauricular incision. The facial nerve was preserved and its function was monitored throughout by direct electrical stimulation. The capsular tumor of the left TMJ was reached. Around the joint, numerous dilated venous vessels were encountered and ligated (suture-ligated) to secure hemostasis. The mass on the lateral surface of the left TMJ capsule was then excised sharply, opening the joint capsule in its distal portion, and the specimen was sent for histopathological examination. The joint capsule was re-approximated with sutures, facial-nerve function was reconfirmed upon stimulation, the wound was closed in layers, and a compressive dressing was applied (Figure 2).

Histopathological examination showed fragments of fibrous connective tissue containing an irregular cystic space lacking an epithelial lining (Figure 3 and Figure 4). The differential diagnosis favored a gelatinous pseudocyst (ganglion). Immunohistochemistry (IHC) was used to refine the diagnosis: pan-cytokeratin (clone AE1/AE3) was negative, arguing against an epithelial-lined (including salivary) cyst, and CD31, performed specifically to exclude a vascular lesion, labeled only the endothelium of small vessels within the wall. The absence of any endothelial or epithelial lining on the inner surface of the cavity excluded a vascular malformation (Figure 5). Taken together, the findings were consistent with a ganglion (pseudocyst) of the TMJ capsule.

The postoperative course was uneventful, with normal wound healing. Facial-nerve function remained intact, mouth opening was normal, and movements of the TMJ were preserved. The patient was reviewed by the operating surgeon on the first postoperative day, at one week for suture removal, at 1, 3, 6, and 12 months, and thereafter at approximately yearly intervals. At every visit, pain was scored on a 0–10 numerical rating scale, the maximal interincisal mouth opening was measured with a millimeter ruler, facial-nerve function was graded on the House–Brackmann scale, and the occlusion and surgical scar were assessed clinically. Throughout follow-up, the pain score remained 0/10, mouth opening stayed within normal limits (greater than 40 mm), facial-nerve function was House–Brackmann grade I, and the occlusion was unchanged from baseline. No otological symptoms developed and, in their absence, formal audiometric testing was not considered necessary. The occlusion was normal and unchanged from the preoperative state (Figure 6). The patient has been followed for 21 months. Control magnetic resonance imaging (MRI) performed 17 months after surgery showed no evidence of recurrence in the TMJ region (Figure 7).

## 3. Materials and Methods

A narrative review of the literature was performed to contextualize the present case. PubMed/MEDLINE, Scopus, and Google Scholar were searched from database inception to 1 July 2026, without a lower date limit, using combinations of the terms “temporomandibular joint”, “TMJ”, “ganglion cyst”, “synovial cyst”, “periarticular cyst”, and “pseudocyst” (for example, (“temporomandibular joint” OR “TMJ”) AND (“ganglion cyst” OR “synovial cyst” OR “periarticular cyst” OR “pseudocyst”)). The reference lists of the retrieved articles and of previous systematic reviews were hand-searched (snowballing) to identify additional cases. Eligible records were English-language case reports, case series, and reviews describing histologically confirmed ganglion or synovial cysts of the TMJ. Excluded were non-English articles without an English abstract, lesions unrelated to the TMJ, and other cystic or tumor-like TMJ pathologies (e.g., synovial chondromatosis, pigmented villonodular synovitis, and aneurysmal or simple bone cysts of the condyle). For each eligible report, the following data were extracted where available: first author and year, patient age and sex, affected side, main clinical presentation, lesion type (ganglion vs. synovial), imaging, treatment and surgical approach, duration of follow-up, and recurrence. The database searches returned 187 records, supplemented by 14 records identified from reference lists and previous reviews. After removal of 69 duplicates, 132 records were screened, of which 71 were excluded based on the title and abstract. A total of 61 full-text articles were assessed for eligibility, and 13 were excluded (not related to the TMJ—5; no histological confirmation—4; non-English without an English abstract—3; other TMJ pathology—1). Overall, 48 reports describing approximately 55 cases were included in the qualitative synthesis. The selection process is summarized in Figure 8, and the most representative reports are compiled in Table 1.

## 4. Discussion

The distinction between ganglion and synovial cysts is fundamentally histological. A ganglion cyst is a pseudocyst: its wall is composed of dense fibrous (collagenous) connective tissue that has undergone mucoid or myxoid degeneration, and it lacks any epithelial or synovial cell lining like in the presented case. On the other hand, a synovial cyst is a true cyst that arises from the synovial membrane and retains a synovial or flattened epithelial lining [2,4,5,13]. In peripheral joints, both lesions are thought to follow mucoid degeneration of connective tissue and/or herniation of synovium, often in the setting of degenerative joint disease or trauma. Only synovial cysts have additionally been linked to a primary inflammatory process [5,14]. In our patient, the fibrous wall devoid of epithelial or synovial cell lining, together with negative cytokeratin and CD31 confined to the vascular endothelium, established the diagnosis of a ganglion and excluded a vascular malformation and an epithelial-lined cyst.

This histological boundary is frequently blurred in practice. In a critical review of synovial cysts, four reported “ganglion” cases were in fact mislabeled synovial cysts, amounting to nearly a quarter of the synovial-cyst literature, an indication of how often the two are conflated when the lining is not carefully assessed [6]. Indeed, when immunohistochemistry is used to identify a synovial lining, periarticular TMJ cysts appear to be more frequently synovial than ganglion [15]. Accurate histological classification matters, because it is the only reliable way to accumulate meaningful data on behavior and outcome. A degenerative mechanism is further supported by the frequent association with condylar remodeling and osteoarthritis: a substantial proportion of TMJ ganglions are accompanied by a deformed or hypertrophic mandibular head [3,16].

TMJ ganglion and synovial cysts show a consistent demographic profile. In the most comprehensive pooled review of 49 ganglion and synovial cysts, lesions occurred with a female-to-male ratio of about 3:1 and a median age of 46 years (range 11–64) [2]. A systematic review restricted to ganglion cysts found a comparable pattern. About 70% were female with a mean age of roughly 49 years [3]. Our patient, a 48-year-old woman, fits this profile closely. The typical presentation is a slowly progressive, often painless preauricular swelling; pain and trismus are reported in a substantial minority (pain in roughly half and trismus in about a third of cases in some series), while malocclusion, tinnitus, aural fullness, and recurrent otological symptoms occur less commonly [2,3,9,17]. Posteriorly located lesions can narrow the EAC and produce position-dependent hearing loss, and rare cases have extended intracranially, presenting with facial-nerve palsy or even seizures mimicking a primary brain tumor [10,11,18]. Recent reports have further underscored dynamic external auditory canal obstruction with position-dependent hearing loss and osteoarthritis-associated presentations [16,19]. In our patient, the lesion was painless with normal mouth opening and intact facial-nerve function. An unremarkable but characteristic presentation that, as is typical, first suggested a parotid neoplasm.

The relationship between TMJ ganglion or synovial cysts and dental occlusion deserves comment, particularly because a mild occlusal cant is visible in the postoperative clinical photograph of our patient (Figure 6a). We found no evidence in the literature that a pre-existing malocclusion causes or predisposes to a TMJ ganglion cyst. The reported etiology is a local mucoid or myxoid degeneration of periarticular connective tissue rather than a malocclusion-driven mechanism [3,5]. Conversely, occlusal change has occasionally been reported as a consequence of the cyst itself, when a sufficiently large or posteriorly located lesion mechanically displaces the condyle and produces an acute, usually unilateral malocclusion, as in the case of unilateral non-occlusion secondary to a TMJ ganglion described by Suhr and Mager [9]. No such mechanism was observed in our patient. The cyst was small and located laterally, it did not displace the condyle, the occlusion was normal preoperatively and remained unchanged after excision, and there was no functional disturbance. The occlusal cant seen in Figure 6a is therefore best interpreted as a pre-existing dental feature unrelated to the cyst. Because the occlusion was stable and asymptomatic, the patient was not investigated with frontal cephalometry or a panoramic radiograph and was not referred for orthodontic assessment, so a dental versus skeletal origin cannot be formally distinguished. More broadly, unilateral degenerative TMJ disorders can be associated with vertical facial asymmetry and occlusal canting, but this has been described for disk displacement and osteoarthritis rather than for periarticular cysts. Given the small number of TMJ ganglion and synovial cysts reported worldwide, any association between these cysts and occlusion or facial symmetry can neither be established nor excluded with confidence, and this remains an open question for future, adequately documented series.

The preoperative differential diagnosis of a preauricular cystic mass is broad and includes parotid tumors, synovial cysts, ganglion cysts, epidermoid/dermoid cysts, synovial chondromatosis, pigmented villonodular synovitis, an intraparotid lymph node, a venous or lymphatic malformation, a facial-nerve schwannoma, and aneurysmal or simple bone cysts of the condyle [2,7]. Of particular practical importance, a TMJ ganglion may closely mimic a cystic salivary lesion, either a non-neoplastic salivary (e.g., salivary-duct) cyst or a salivary neoplasm with cystic change such as Warthin tumor or (low-grade) mucoepidermoid carcinoma so that, as in our patient, the lesion is frequently worked up as a suspected parotid tumor [20,21]. Ultrasound, CT, and MRI each contribute. Ultrasonography is inexpensive, non-invasive, and radiation-free and can demonstrate a superficial cystic lesion or joint effusion, and although less sensitive than cone beam computed tomography (CBCT), it may help screen for degenerative and condylar changes [22]. CT and CBCT define bony relationships and any condylar remodeling and typically show a well-defined, hypodense, non-enhancing fluid collection, while MRI best characterizes the cystic nature (T2-hyperintense, non-enhancing, or thin rim-enhancing) and the relationship to the joint space [2,23]. In our patient, because the tumor was initially interpreted as a parotid neoplasm, CT was the first and, as it turned out, the only preoperative cross-sectional study. MRI was not obtained before surgery. Even so, imaging is frequently indeterminate, as in our case, and cannot reliably separate a ganglion from a synovial cyst. Fine-needle aspiration, yielding clear gelatinous fluid, can support the diagnosis and is advocated by some as part of the workup. Ultrasound guidance can improve the accuracy of such aspiration and of minimally invasive TMJ procedures more generally, and in one series, a synovial cyst even resolved after puncture alone [2,17,24]. Ultimately, histopathological examination of the excised lesion remains essential for definitive diagnosis.

Complete surgical excision through a preauricular approach remains the treatment of choice for symptomatic lesions [2,3,12]. The principal technical challenges are protection of the facial nerve, which lies nearly in the operative field, and complete removal of a thin-walled cyst that is often densely adherent to the capsule and prone to intraoperative rupture. In a review that specifically addressed the surgical approach, 24 of 39 cases were excised via a preauricular route, and intraoperative cyst rupture occurred in 8 of those 24, prompting some authors to favor curettage combined with discectomy when the wall cannot be dissected intact [12]. When posterior extension involves the EAC, TMJ arthroplasty with reconstruction (for example, an autogenous tragal cartilage graft) has been used successfully, and condylar recontouring may be added where osteoarthritis and condylar hypertrophy coexist [3,25].

For asymptomatic lesions or patients unfit for or declining surgery, conservative management, observation, or aspiration is reasonable, recognizing that aspiration alone carries a higher chance of recurrence than complete excision [17]. Because these lesions are probably developmental/degenerative rather than neoplastic, routine long-term surveillance for the cyst itself has been considered unnecessary by some authors [2]. Recurrence of TMJ ganglion and synovial cysts has traditionally been regarded as very rare: pooled reviews reported no recurrences and correspondingly short follow-up, with a median of only about 10 months [2,6]. That reassuring picture has since been tempered. A case series documented a recurrent ganglion cyst 3.5 years after complete initial excision, with two new cystic lesions at the original site, explicitly cautioning that the short follow-up of most reports probably underestimates the true recurrence rate [17]. In a case of intracranial extension, the lesion regrew five years after the first operation and required reoperation [11]. Systematic reviews restricted to ganglion cysts likewise cite a low but non-zero recurrence (on the order of 10%), typically attributed to incomplete removal [3]. It is also worth noting that successful excision does not guarantee symptom relief. In one synovial-cyst case, joint pain persisted despite cyst removal and four years without radiological recurrence [6].

These observations support longer, imaging-based follow-up rather than the brief clinical review that has characterized much of the literature. Our patient has been followed for 21 months, with control MRI at 17 months confirming the absence of recurrence. A follow-up interval and an objective imaging endpoint compare favorably with most published reports, though still short of the ~3.5–5-year window in which the documented recurrences appeared. Accordingly, we plan continued follow-up with clinical review and repeat MRI at approximately yearly intervals, aiming to extend objective surveillance across and beyond this at-risk period.

Table 1 summarizes representative reports of TMJ ganglion and synovial cysts alongside the present case. Across these reports, the pattern is consistent: a middle-aged, predominantly female patient with a unilateral preauricular swelling, an indeterminate cystic lesion on imaging, a histological diagnosis obtained only after excision, treatment by preauricular excision under general anesthesia, and an uneventful outcome in the majority of cases.

**Table 1:** Representative reported cases and reviews of temporomandibular joint (TMJ) ganglion and synovial cysts compared with the present case. All cases were histologically confirmed.

Study (Year)	Age/Sex	Side	Size (mm)	Main Presentation	Type	Imaging	Treatment/Approach	Follow-Up	Recurrence
Reychler et al. (1983) [26]	NR	Unilat.	NR	Preauricular swelling	Synovial	NR	Excision	NR	None
Shiba et al. (1987) [27]	NR	Unilat.	NR	Preauricular swelling	Ganglion	NR	Excision	NR	None
Copeland & Douglas (1988) [28]	NR	NR	NR	Preauricular mass	Ganglion	NR	Excision	NR	None
el-Massry & Bailey (1989) [29]	NR	NR	NR	Preauricular swelling	Ganglion	NR	Excision	NR	None
Tom et al. (1990) [23]	NR/F	Bilat.	NR	Bilateral swelling	Ganglion	CT + MR	Imaging study	NR	NR
Farole & Johnson (1991) [30]	NR	Bilat.	NR	Bilateral preauricular swelling	Synovial	CT	Excision	NR	None
Chang et al. (1997) [31]	NR	Unilat.	NR	Preauricular swelling	Gangl. + syn. (2)	CT	Excision	NR	None
Silva et al. (2005) [32]	NR/F	Unilat.	NR	Preauricular swelling	Ganglion	CT	Preauricular excision	NR	None
Mumert et al. (2012) [10]	Adult	Unilat.	NR	VII palsy; intracranial ext.	Ganglion	CT + MRI	Resection	NR	NR
Savolainen & Kellokoski (2013) [7]	56/F	R	NR	Pain + preauricular prominence	Ganglion	CT + MRI	Preauricular excision	6 mo	None
Suhr & Mager (2013) [9]	Adult	Unilat.	NR	Unilateral non-occlusion	Ganglion	MRI	Excision	NR	None
Lee et al. (2014) [5]	Adult/F	Unilat.	NR	Preauricular mass	Ganglion	CT + MRI	Preauricular excision	NR	None
Steen & Hofstede (2015) [2]	Median 46	—	NR	Preauricular swelling ± pain	Review, n = 49	CT/MRI	Excision (GA)	~10 mo	None reported
Zheng et al. (2015) [33]	24/M	Unilat.	NR	TMD-like symptoms	Ganglion	MRI	Surgical excision	NR	None
Levarek & Nolan (2016) [34]	Adult	Unilat.	NR	Preauricular mass	Ganglion	CBCT + MRI	Subtotal excision	NR	Resolved
Buckley et al. (2019) [35]	NR	Bilat.	NR	Bilateral preauricular swelling	Synovial	MRI	Excision	NR	None
Maribo et al. (2019) [6]	52/F	Unilat.	NR	TMJ pain, reduced opening	Synovial	MRI	Excision	4 y	None (sympt. persisted)
Segami & Nojima (2019) [12]	55/M	L	25	Preauricular swelling, restricted movement	Ganglion	MRI	Preauricular excision → curettage + discectomy	NR	None
Kamer et al. (2020) [11]	Adult/M	Unilat.	NR	Seizures; intradural ext.	Ganglion	CT + MRI	Resection + dural repair	5 y	Regrowth at 5 y
Nys et al. (2020) [17]	Mean 44	R > L	NR	Preauricular swelling/pain; otologic	Series n = 6	CT/MRI	Excision/puncture/obs.	Up to years	1 recurrence (3.5 y)
Krishnan et al. (2023) [3]	Mean ~49	—	NR	Preauricular swelling	Review, n = 20	CT/MRI	Excision	Variable	~10%
Moreira et al. (2026) [25]	63/M	L	NR	Aural fullness, hearing loss; EAC herniation	Ganglion	CT + MRI	Preauricular excision + tragal graft	1 y	None
Akbar et al. (2026) [19]	72/F	L	24 × 21 × 10	Position-dependent hearing loss; EAC obstruction	Ganglion	MRI	Preauricular excision + partial discectomy	2 y	None
**Present case (2025)**	**48/F**	**L**	**15 × 10 × 7**	**Painless preauricular swelling; condylar hypertrophy**	**Ganglion**	**CT**	**Neuronavigation-guided preauricular excision**	**21 mo (MRI 17 mo)**	**None**

Abbreviations: F, female; M, male; R, right; L, left; NR, not reported; GA, general anesthesia; EAC, external auditory canal; TMD, temporomandibular disorder; MRI, magnetic resonance imaging; MR, magnetic resonance; Nav, intraoperative neuronavigation; obs., observation; sympt., symptoms.

### Limitations

Several limitations should be acknowledged. As a single case report, the generalizability of our observations is inherently limited. Although the follow-up is longer and better documented than in most previous reports, it remains shorter than the 3.5–5-year interval at which recurrences have been described, so late recurrence cannot be excluded. The findings reflect a single-surgeon, single-center experience and were collected retrospectively. In addition, MRI was not obtained preoperatively, because the mass was initially managed as a suspected parotid tumor, limiting soft-tissue characterization and assessment of any communication with the joint space.

## 5. Conclusions

Ganglion cysts of the TMJ are rare, benign pseudocysts that mimic parotid and other preauricular lesions that are diagnosed definitively only on histology, where the absence of an epithelial or synovial cell lining separates them from the true synovial cyst. Complete excision through a preauricular approach with careful facial-nerve preservation remains the treatment of choice. In practical terms, several points emerge from this case and the reviewed literature. A TMJ ganglion cyst should be included in the differential diagnosis of any swelling or tumor in the preauricular or parotid region. Cross-sectional imaging (CT, CBCT, or MRI) can characterize the lesion and its relationship to the joint but cannot reliably distinguish between a ganglion and a synovial cyst. A definitive diagnosis can only be made by histology, which shows the absence of an epithelial or synovial lining. Furthermore, functional outcomes such as mouth opening, occlusion, and facial-nerve function are usually unaffected, as was observed in the present case. Additionally, as late recurrences have been documented three to five years after excision, objective, imaging-based surveillance should extend across this at-risk period.

## Figures and Tables

**Figure 1:**
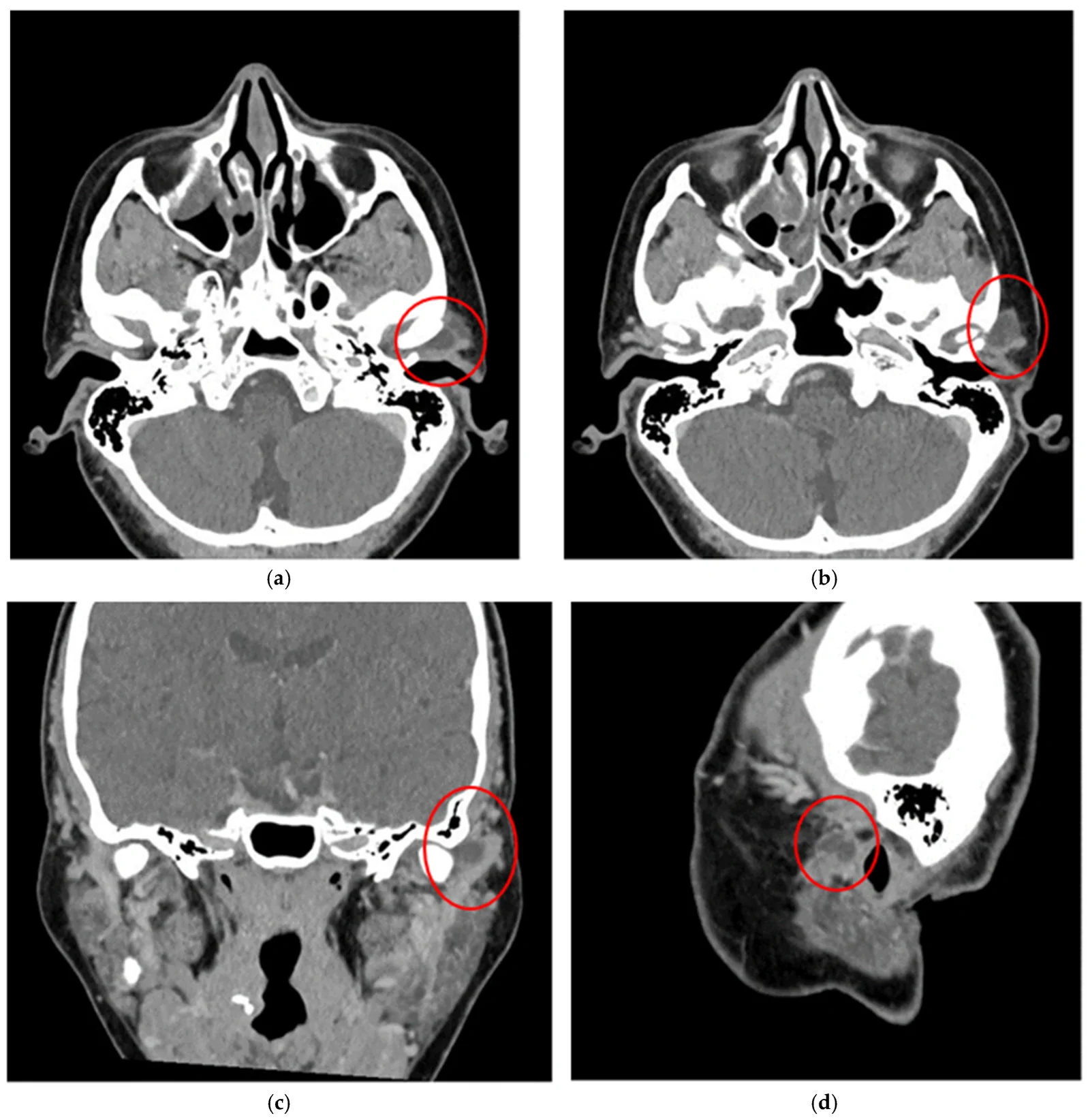
Preoperative contrast-enhanced computed tomography of the left temporomandibular joint. (**a**,**b**) Axial soft-tissue windows at the level of the external auditory canal show a well-defined, hypodense (fluid-attenuation, ~15 HU) lesion lateral to the left condyle, abutting the anterior wall of the external auditory canal. (**c**) Coronal and (**d**) sagittal scans demonstrate the relationship of the non-enhancing lesion to the condylar process. Lesion dimensions were 15 × 10 × 7 mm. Red circles indicate the cystic lesion adjacent to the left mandibular condyle.

**Figure 2:**
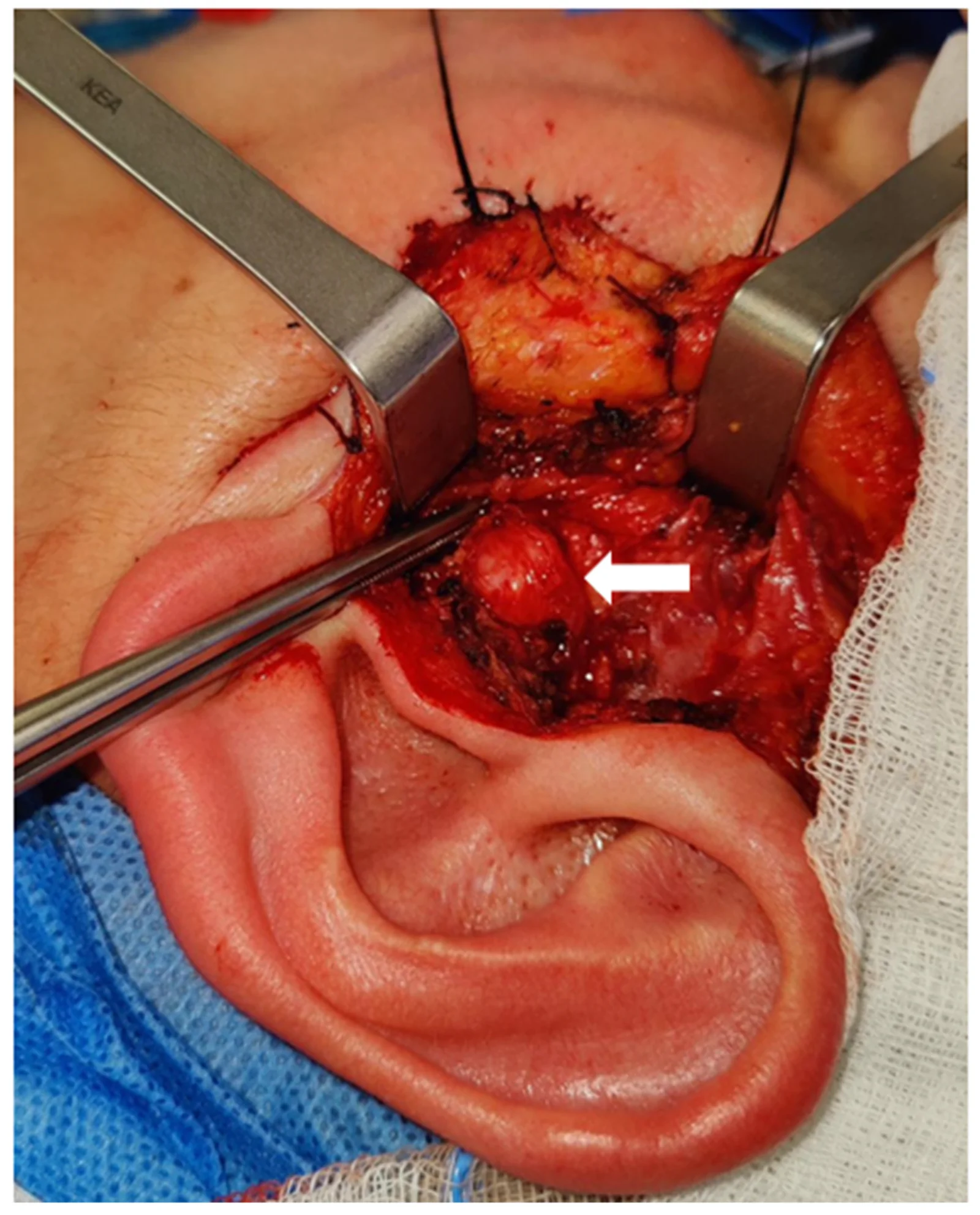
Intraoperative view presenting the tumor of the left TMJ capsule. The arrow indicates the ganglion cyst on the lateral surface of the left temporomandibular joint capsule.

**Figure 3:**
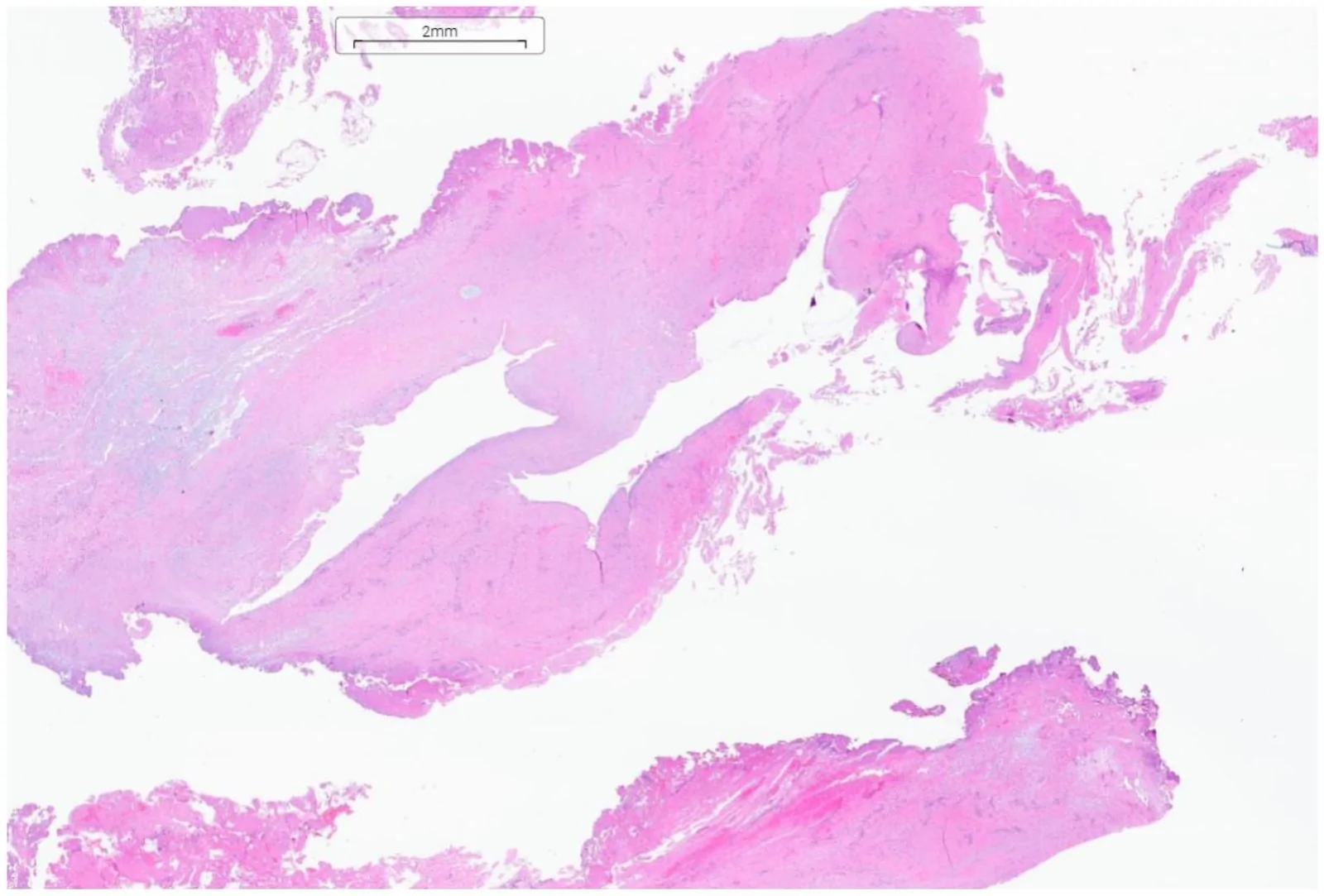
Low-power photomicrograph showing an irregular cystic structure with a thick fibrous wall. Hematoxylin and eosin (H&E), original magnification ×60.

**Figure 4:**
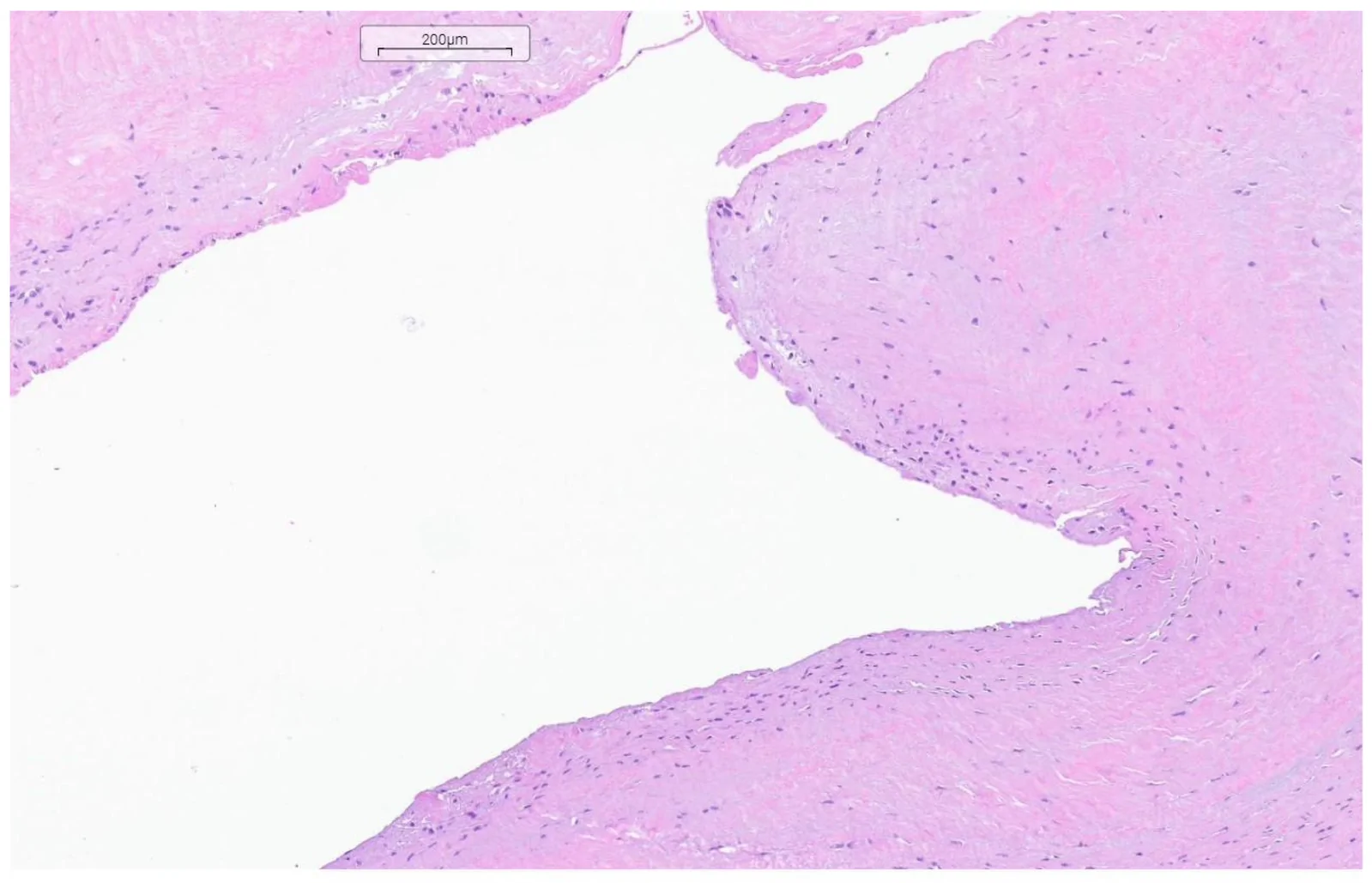
The fibrous wall of the cystic structure lacks a synovial or epithelial cell lining—the histological hallmark distinguishing a ganglion (pseudocyst) from a true synovial cyst. H&E, original magnification ×140.

**Figure 5:**
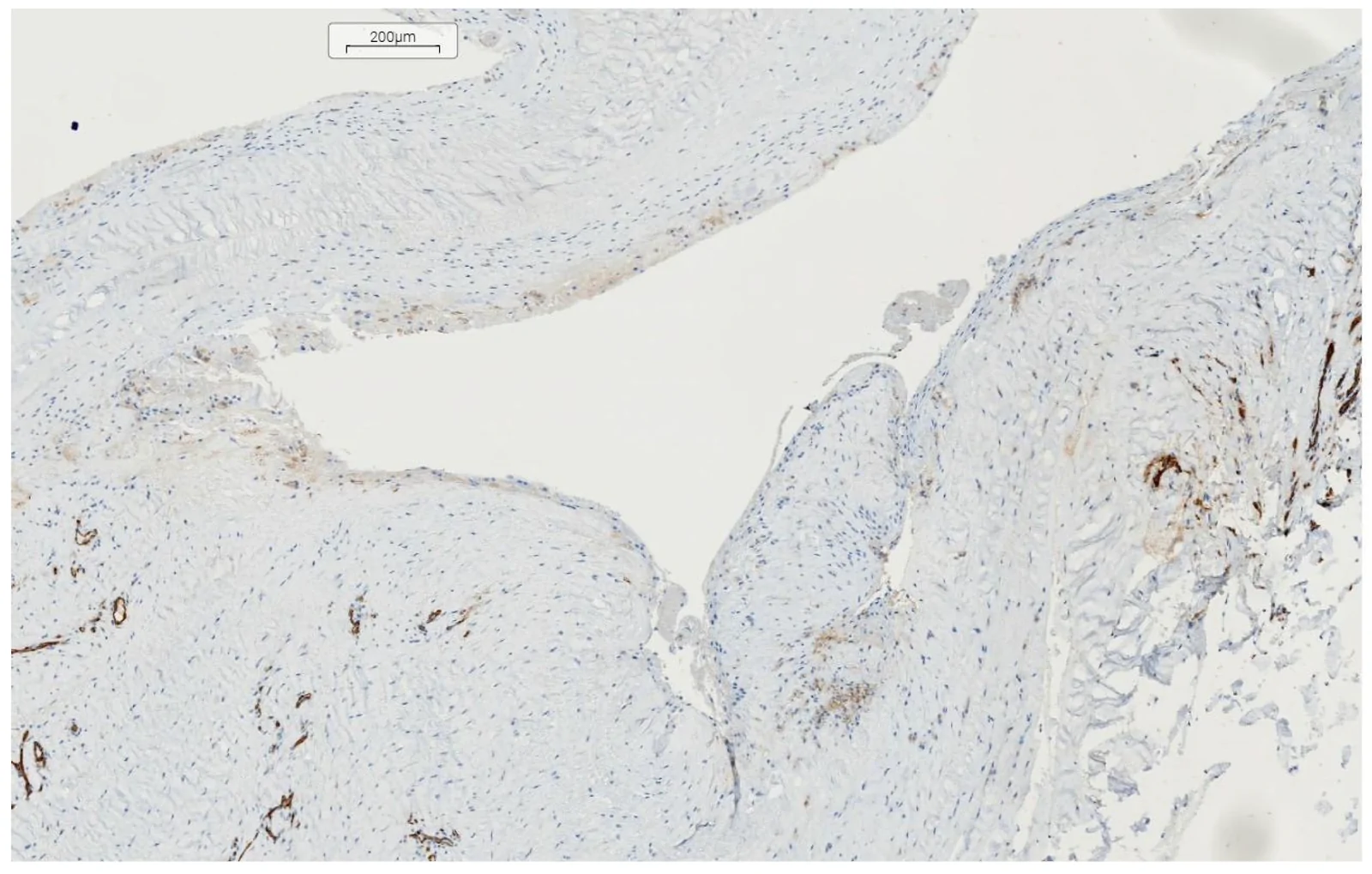
The absence of an endothelial layer on the inner surface of the irregular cyst excludes a vascular malformation; CD31 highlights only the endothelium of small vessels within the wall. Immunohistochemistry, CD31 (clone JC70A, Roche/Ventana), original magnification ×140.

**Figure 6:**
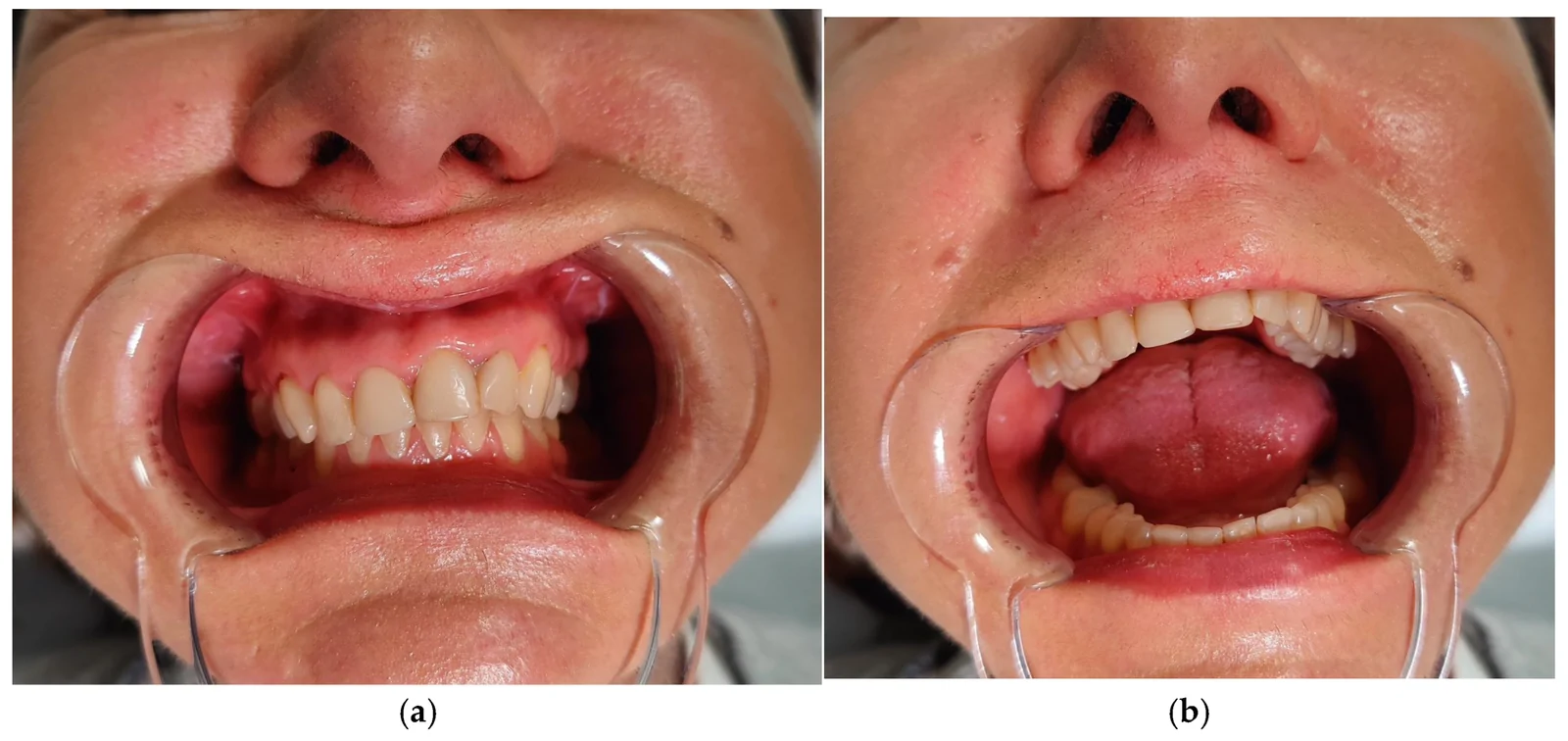
Postoperative clinical view 15 months postoperatively demonstrating a stable, physiological occlusion (**a**), unchanged from the preoperative status with proper mouth opening (**b**).

**Figure 7:**
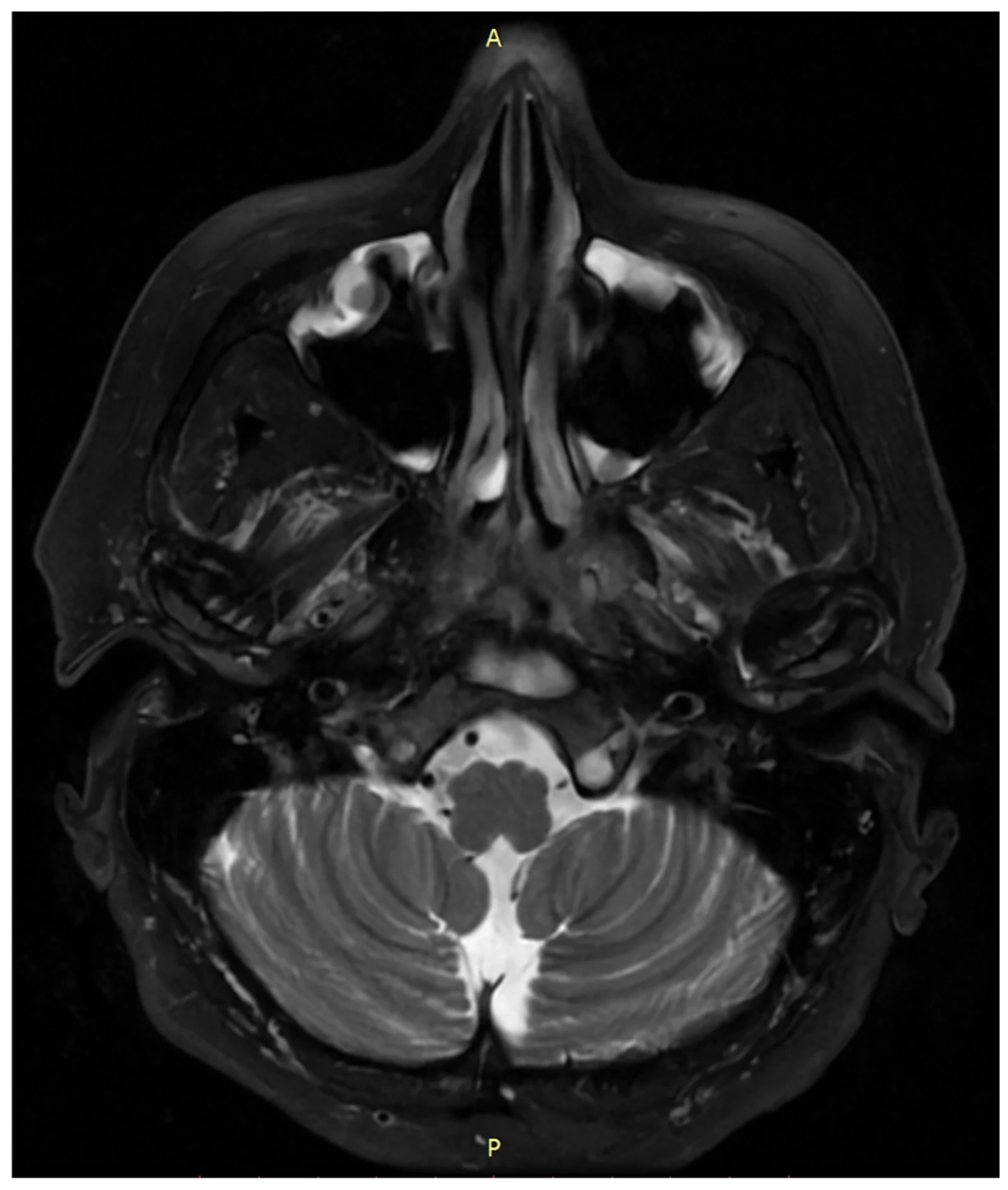
Follow-up MRI obtained 17 months after surgery. Axial T2-weighted image showing no evidence of recurrence in the left temporomandibular joint region.

**Figure 8:**
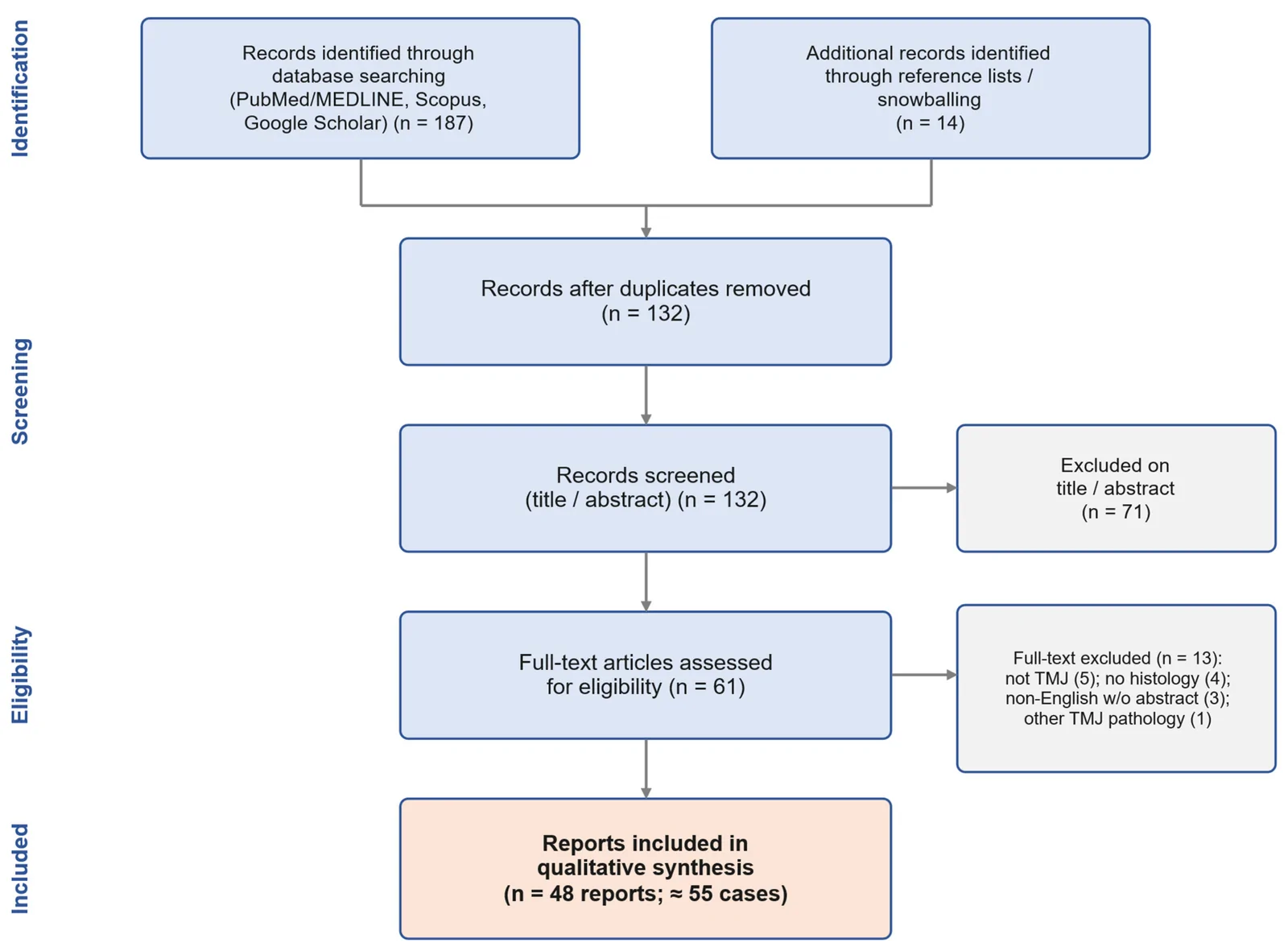
Flowchart diagram of the identification and selection of studies for the literature review.

## Data Availability

The data supporting the findings of this case report are available from the corresponding author upon reasonable request, subject to maintaining patient confidentiality.

## Abbreviations

The following abbreviations are used in this manuscript:

TMJ	Temporomandibular joint
EAC	External auditory canal
CT	Computed tomography
MRI	Magnetic resonance imaging
HU	Hounsfield units
IHC	Immunohistochemistry
H&E	Hematoxylin and eosin
FNA	Fine-needle aspiration
